# Hepatic arterial infusion chemotherapy plus regorafenib in advanced colorectal cancer: a real-world retrospective study

**DOI:** 10.1186/s12876-022-02344-4

**Published:** 2022-07-04

**Authors:** Guang Cao, Xiaodong Wang, Hui Chen, Song Gao, Jianhai Guo, Peng Liu, Haifeng Xu, Liang Xu, Xu Zhu, Renjie Yang

**Affiliations:** 1 Key Laboratory of Carcinogenesis and Translational Research (Ministry of Education, Beijing), Department of Interventional Therapy, Peking University Cancer Hospital and Institute, Beijing, 100142 China; 2 Department of Interventional Therapy, National Cancer Center/National Clinical Research Center for Cancer/Cancer Hospital, Chinese Academy of Medical Sciences and Peking Union Medical College, Beijing, 100021 China

**Keywords:** Colorectal cancer, Hepatic arterial infusion, Liver metastasis, Regorafenib, Survival

## Abstract

**Background:**

Hepatic arterial infusion chemotherapy delivers the drug directly to the liver. We aim to explore the benefits and tolerability of Hepatic arterial infusion chemotherapy plus regorafenib in advanced colorectal liver metastasis refractory to standard systemic chemotherapy.

**Methods:**

This study analyzed 47 patients treated with hepatic arterial infusion chemotherapy plus regorafenib after standard systemic oxaliplatin and/or irinotecan in combination with bevacizumab or cetuximab between Jan 2017 and Jun 2020. Regorafenib was given for only 3 weeks in a 4-week cycle.

**Results:**

Among 47 patients, 32 (68%) were males. The median age was 61 (29–75). With a median follow-up of 22.2 months (3.7–50.7 months). Before Hepatic arterial infusion chemotherapy administration in combination with regorafenib, 34 (72.3%) patients previously received ≥ 2 prior lines of systemic therapy and 37 (78.7%)patients previously received targeted biological treatment (anti-VEGF or anti-EGFR, or both). The initial doses of regorafenib were 40 mg/d (n = 1, 2.13%), 80 mg/d (n = 11, 23.43%), 120 mg/d (n = 2, 4.26%), and 160 mg/d (n = 23, 48.94%), while for 24.6% (n = 14) dose was unknown. Median Overall Survival was 22.2 months. Median Progression-Free Survival was 10.8 (95% CI: 9.0–13.7) months. Common Adverse Events were hand-foot skin reaction (12.77%), fatigue (6.38%), vomiting (6.38%), and decreased appetite (6.38%). Only 2 patients discontinued regorafenib due to Adverse Events.

**Conclusions:**

Regorafenib combined with Hepatic arterial infusion was effective and tolerable in patients with liver predominant metastasis of colorectal cancer. Hence, this therapy can be considered as an alternative for second- or subsequent lines of therapy in patients refractory to standard systemic chemotherapy.

## Introduction

Colorectal cancer (CRC) is the third most prevalent malignant disease and is the second leading cause of cancer-related deaths worldwide according to the estimates from GLOBOCAN 2020 [1, 2]. Almost 20% of the patients with CRC harbor hepatic metastases at the time of diagnosis [3], whereas around 50% of the CRC patients develop metastasis of the liver during the clinical course of the disease [3, 4]. A large proportion (approximately 75–90%) of metastatic CRC patients present with unresectable liver disease which normally reports a 5-year overall survival (OS) of 6–10% [5–8]. Thus, in such patients, tumors of the liver can be downsized and/or converted to resectable colorectal liver metastasis (CRLM) from the unresectable form with systemic or liver-directed chemotherapy also known as hepatic arterial infusion chemotherapy (HAIC) [9–11]. Numerous studies have outlined the utility of HAIC with or without systemic therapy in significantly prolonging the progression-free survival (PFS) when compared to the systemic therapy alone (or with no further therapy) [10, 11]. Above all, HAIC may have a definitive “salvation” role in the treatment of patients with predominant liver or liver only metastasis in advanced CRLM following the failure from first/second-line systemic therapy [12, 13]. In addition, patients with advanced-stage and/or chemo-refractory CRC often cannot undergo radical treatment due to the status of liver function, shortage of residual liver volume, and hepatic metastasis [14].

Since the late 1980s, HAIC is being exhaustively studied in patients with CRLM. Since the hepatic artery provides most of the blood supply towards CRLM compared to the portal veins, this serves as the biological rationale behind HAIC [15]. As the name suggests, the main feature of HAIC is the direct delivery of chemotherapeutic agents as an infusion into the hepatic artery through a surgically or percutaneously implanted catheter that is connected to a hepatic arterial port/external pump. This consequently allows preferential drug delivery directly to CRLM with relative sparing of the background liver parenchyma and allows drugs to circumvent the first-pass effects of hepatic excretion. Thus, the tumor cells are exposed to a significantly higher concentration of chemotherapeutic agents while suppressing the systemic toxic effects [16].

Regorafenib (Bay 73-4506) is an oral multi-kinase inhibitor that exerts its anti-tumor effect by blocking and inhibiting the activity of multiple protein kinases involved in tumor angiogenesis, tumorigenesis, metastasis, and tumor immunity [17]. The efficacy of regorafenib was first observed in a phase III CORRECT trial, where treatment with regorafenib offered survival benefits to patients with treatment-refractory metastatic CRC (median overall survival: 6·4 months in regorafenib group vs. 5·0 months in placebo group, HR 0·77; 95% CI 0·64–0·94, one-sided *P* = 0·0052) [18]. Later, in an international, multicenter, placebo-controlled phase III CONCUR trial [NCT01103323] [19] patients with metastatic CRC that had treatment failure to either standard therapy or prior two lines of treatment showed a higher overall survival with regorafenib than placebo (8.8 months in regorafenib only vs. 6.3 months in the placebo group; HR 0.55, 95% CI 0.40–0.77, one-sided *P* = 0.00016), however, 97% of the patients in the regorafenib group had drug-related adverse events (AEs). Although this study showed favorable outcomes with regorafenib, in previously treated subgroups, there is a possibility of confounding error owing to the small sample size and due to few patients receiving follow-up treatments (32% in the regorafenib group and 54% in the placebo group with previous treatment with anti-VEGF-targeted treatment received subsequent systemic therapy) [19].

Therefore, regorafenib has become the standard recommendation according to the NCCN guidelines (Version 2.2021) as an additional line of therapy for patients with advanced or metastatic CRC, particularly for those who are refractory to chemotherapy [20]. A real-world study conducted in CRLM patients’ refractory to standard chemotherapy in China further supports this. A longer OS (16.7 months) in the group that continued regorafenib treatment (n = 20) compared with those who stopped regorafenib (n = 52) with a median OS of 9.1 months (*P* = 0.116) [21]. Moreover, regorafenib is also suggested as a third-line option after treatment with cetuximab as it yields a longer OS than the reverse sequence of cetuximab following regorafenib [22, 23].

Recently, Cao et al. showed that the regorafenib plus drug-eluting-beads-TACE group yielded a higher median PFS than the regorafenib-only group (7.6 vs. 4.1 months, *P* < 0.001, respectively) in CRLM that has not responded to the standard treatment regimens. The median OS was also higher in the regorafenib plus drug-eluting-beads-TACE group versus the regorafenib-only group (15.7 vs. 9.2 months, *P* < 0.001) [24]. Many studies have confirmed the efficacy of tyrosine kinase inhibitors (TKI) such as regorafenib and sorafenib combined with HAIC in the localized treatment strategies of TACE in hepatocellular carcinoma [25–28]. However, an evolving perspective of using regorafenib as third-line and later-line treatment of patients with metastatic CRC is shared by the experts, provided that the dose is modified to manage toxicity while improving the quality of life [29]. Thus, due to the aforementioned clinical plausibility of the “HAI-REGO” (HAIC-Regorafenib) combination, we hypothesized that the use of second-line regorafenib in conjunction with HAIC might have a better outcome, particularly a longer OS in CRC patients experiencing treatment failure to first/second-line systemic therapy. Based on this rationale, this single-center exploratory study was conducted to assess the efficacy and tolerability of HAIC combined with second-/third-line regorafenib in patients with predominant liver metastasis.

## Materials and methods

### Study design and setting

This was a real-world, retrospective, single center, review study that evaluated the efficacy and safety of HAIC combined with Regorafenib in Chinese CRC patients with liver predominant liver metastasis. The data was reviewed from the medical records of the patients at one of the hospitals from June 2017 to October 2020. The study was conducted by the Declaration of Helsinki (as revised in 2013) and with the ethical approval from the Ethics Committee of Peking University Cancer Hospital and received Institutional Review Board approval. Due to the study’s retrospective nature, the provision of informed consent was waived off by the Ethics Committee of Peking University Cancer Hospital.

All the data was collected from the electronic health records, including age, sex, body mass index (BMI), complications of extrahepatic spread, carcinoembryonic antigen (CEA), number of intrahepatic lesions/percentages of liver involved, maximum lesion diameter, and previous systemic treatments. The treatment-related variables of interest were dosage adjustment, course of treatment, reasons for withdrawal, duration of treatment, and frequency of HAIC, and reasons for the termination of treatment. The last follow-up was held on June 15, 2020.

### Participants

All participants received at least one treatment cycle of HAIC combined with regorafenib and had a histological diagnosis of colorectal adenocarcinoma with liver metastasis. The study also enrolled cases with primary colorectal carcinoma either previously resected or not, given that the liver was the dominant site of metastasis. Patients with liver metastasis who experienced therapeutic failure and/or intolerance towards prior FOLFOX and/or FOLFIRI combined with cetuximab and/or bevacizumab were also eligible for the study. Few cases are CRC with heavy liver metastasis without prior standard system treatment were also included in this study.

The inclusion criteria consisted: (1) > 18 years of age; (2) pathologically or clinically diagnosed CRC with liver predominant metastasis (3) unresectable or refused surgery; (4) first and/or second-line line system chemotherapy treatment; (5) Eastern Cooperative Oncology Group (ECOG) performance status (PS) score of 0–2; (6) Child–Pugh grade A or B; (7) underwent HAIC combined with regorafenib.

**Fig. 1:**
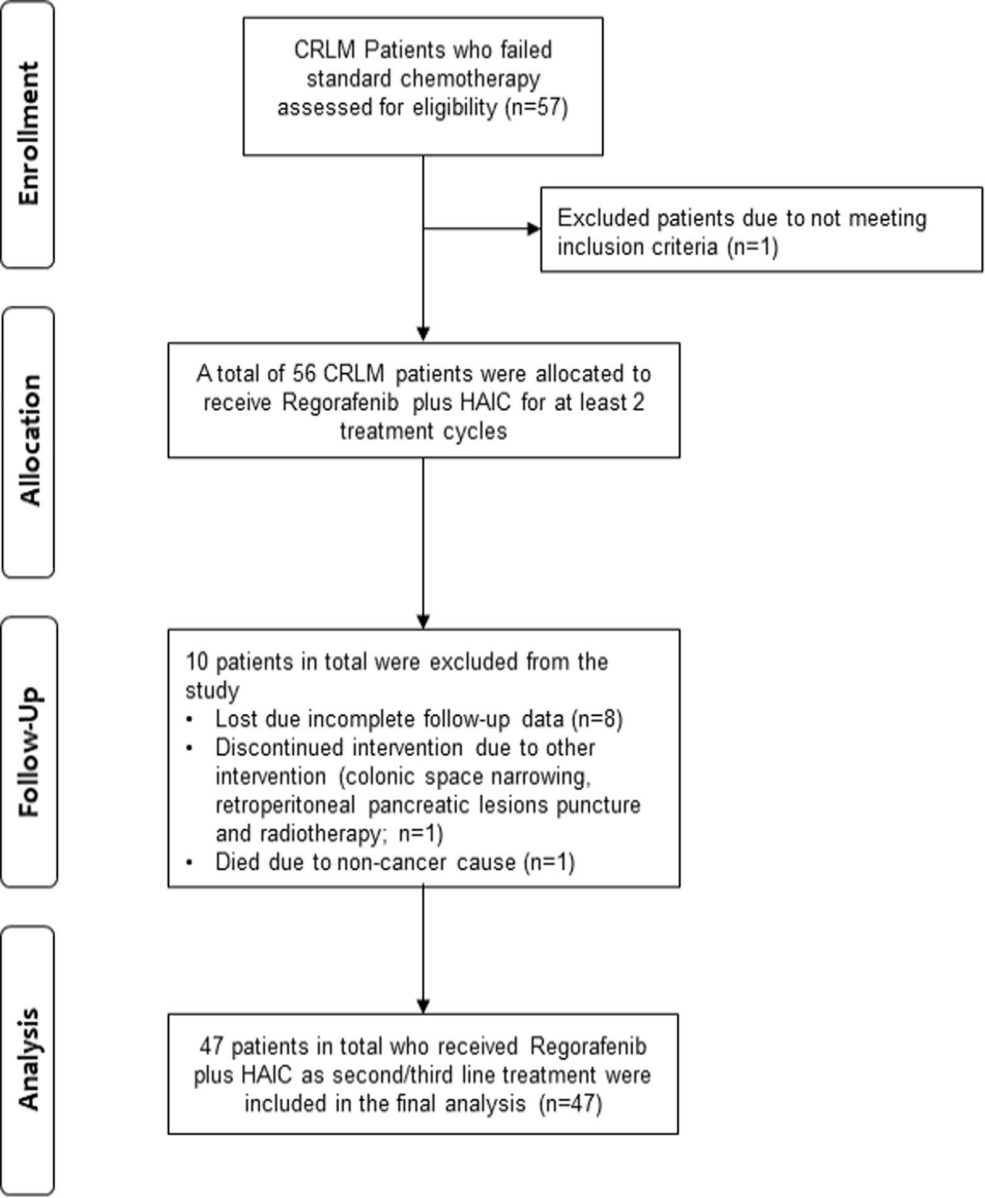
Patient disposition flowchart

The exclusion criteria were: (1) incomplete data; (2) co-morbid diseases; (3) history of other malignant tumors; (4) pregnant or lactating women, or (5) participation in a clinical trial. Figure 1 provides the disposition of the total CRLM patients included in the final analysis.

### Procedures and treatment

A 5 French temporary catheter was inserted into the hepatic artery via the femoral artery using standard interventional radiology techniques. After insertion, the 2.6/2.7F micro-catheter tip was indwelled in the hepatic artery proper also known as the proper hepatic artery. Extrahepatic arteries such as the right gastric artery and accessory left gastric artery had to be occluded by micro-coils. The functionality of the system was examined with the digital subtraction hepatic angiography and cone-beam computed tomography (CBCT) hepatic angiography. The intra-arterial chemotherapy consisted of 50 mg/m^2^ infusions of oxaliplatin for 2 h (Jiangsu Hengrui Medicine Co, Ltd., China) followed by 1000 mg/m^2^ of 5-fluorouracil (Jiangsu Hengrui Medicine Co, Ltd., China) for 22 h on day 1–2 every 4–5 weeks. For each cycle, leucovorin calcium 200 mg/m^2^ was intravenously administered for 2 h from the initiation of 5-fluorouracil infusion. For patients aged 75 years and above, 20% of the dose was tapered. Antiemetic prophylaxis was achieved with 8 mg 5-HT3 antagonist given intravenously daily on days 1–3. The catheter and the micro-catheter would all be removed once the regimen was over. Noteworthily, regorafenib Following this break, patients started taking regorafenib for 3 weeks in every 4-week cycle. The starting dose of regorafenib was at physician’s discretion based on the patients’ treatment status. Regorafenib treatment was initiated only 5 days after the patients had received HAIC and were discontinued for 2 days before the next HAIC.

### Outcome measures

The primary endpoints of the study were PFS defined as time elapsed between treatment initiation and tumor progression or death from any cause, Time to Progression (TTP) theoretically differing from PFS in that the event of interest was only tumor progression, and OS, calculated from the first HAIC treatment. The PFS was defined as the time from the first time of HAIC or regorafenib introduction to the occurrence of disease progression or death from any cause, while OS was defined as the time from the first HAIC or regorafenib introduction to the occurrence of death from any cause.

The secondary endpoints were percentage of patients with complete response (CR), partial response (PR), progressive disease (PD), stable disease (SD), objective response rate (ORR), and disease control rate (DCR) both assessed by Modified Response Evaluation Criteria in Solid Tumors (mRECIST), drug safety (by CTCAE 5), and surgical complications (by Clavien-Dindo classification). Response rate (RR) was evaluated every 6 weeks and AE was graded according to the National Cancer Institute Common Toxicity Criteria version 5.

### Statistical analysis

The continuous variables with a normal distribution were expressed as mean ± standard deviations, and those with a skewed distribution were expressed as medians (ranges). Categorical variables were expressed as n (%). Survival analysis was performed using the Kaplan–Meier method and the log-rank test for determining P-values. The prognostic factors were analyzed using the Cox proportional hazards models. Patients with missing data or dropping out of follow-up were omitted from the final analysis.

## Results

### Baseline characteristics

A total of 57 patients were found to meet the study criteria, however, information on therapy before regorafenib was missing in 7 (12%) patients, and 3 patients due to incomplete follow-up data were excluded from the final analysis. A total of 47 patients were included in the study with most of the participants 32 (68%) being males as shown in Table 1. The median follow-up was 15.6 months (range 1.5, 50.7 months) and the median age was reported at 60 years (range 29, 75 years). Figure 1 illustrates the disposition of patients as a flow diagram.

**Table 1:** Baseline characteristics and distribution of the sample population

Characteristics	Median (min., max.)	IQR (Q1, Q3)
Age (years)	60 (29, 75)	52, 64
Follow-up*	15.6 (1.5, 50.7)	(9.2, 22.5)
Gender	Number of cases	Percentage (%)
Male	40	70.2
Female	17	29.8
Total	57	100.0
Primary tumor		
TX	0	0
T0	0	0
Tis	0	0
T1	0	0
T2	2	3.5
T3	24	42.1
T4	10	17.5
Unknown	21	36.8
Regional lymph nodes		
NX	1	1.7
N0	6	10.5
N1	15	26.3
N2	12	21.0
Unknown	23	40.3
Metastasis		
M0	1	1.7
M1	9	15.8
Unknown	47	82.4
ECOG score		
0	15	26.3
1	9	15.8
2	32	56.1
Clinical stage		
cTNM frequency	5	8.8
pTNM frequency	30	52.6
Missing	22	38.6
Total	57	100
Gene types		
KRAS	39	–
NRAS	29	–
BRAF	44	–
EGFR	35	–
RAS status		
NRAS wild type	24	–
KRAS wild type	19	–
KRAS mutation	20	–
Undetected/unknown	100	–
Location of primary lesion		
Rectum	17	29.8
Sigmoid colon	22	38.6
Splenic flexure of colon	2	3.5
Ileocecal part	1	1.7
Rectum + sigmoid colon	2	3.5
Others	11	19.3
Missing	2	3.5
Total	57	100
Location of tumor metastasis		
Lung	1	1.7
Liver	21	37.0
Others	2	3.5
Lung + liver	3	5.3
Liver + others	2	3.5
Missing	28	49.1
Total	57	100
Pre-regorafenib treatment line		
1	13	22.8
≥ 2	37	64.9
Missing	7	12.3
Total	57	100
Previous treatment regimens		
VEGFR & EGFR inhibitor	8	14.3
Anti-EGFR only	13	23.2
Only anti-VEGFR	12	21.4
^#^Others	23	41.1
Total	56	100.0
Treatment prior to regorafenib		
mFOLFOX6	13	22.8
XELOX	27	47.4
FOLFIRI	5	8.8
XELIRI	1	1.7
FOLFOXIRI	1	1.7
Others	10	17.5
Total	57	100
Initial dose of regorafenib in (mg)		
40	1	1.8
80	12	21.1
120	2	3.5
160	28	49.1
Missing	14	24.6
Total	57	100.0
Intrahepatic evaluation after 1st administration of HAIC + regorafenib		
PR	20	35.1
SD	20	35.1
Missing	17	29.8
Total	57	100.0
ORR	(20/40)	50.0
DCR	(40/40)	100.00
Extrahepatic evaluation after 1st administration of HAIC + regorafenib		
PR	4	7.0
SD	7	12.3
PD	7	12.3
Other	12	21.1
Missing	27	47.4
Total	57	100.0
ORR	(4/30)	13.3
DCR	(11/30)	36.7

*DCR* disease control rate, *EGFR* endothelial growth factor receptor, *HAIC* hepatic arterial infusion chemotherapy, *ORR* objective response rate, *PD* progressive disease, *PR* partial response, *SD* stable disease, *VEGFR* vascular endothelial growth factor receptor

*The starting time of calculation is 1stHAIC-start time

^#^oxaliplatin + fluorouracil, capecitabine, capecitabine + oxaliplatin

### Treatment profile of HAIC combined with regorafenib

Patients were treated with HAIC, for a median of 2 (range: 2, 8) sessions. The initial doses of regorafenib were 40 mg/d (n = 1, 1.8%), 80 mg/d (n = 12, 21.1%), 120 mg/d (n = 2, 3.5%), and 160 mg/d (n = 28, 49.1%) while for 24.6% (n = 14) dose was unknown. During the course of treatment, 12.3% patients stopped regorafenib because of progression (n = 7), intolerance (n = 8, 14.01%), and others (n = 12, 21.1%). Due to the coronavirus disease (COVID-19) pandemic, follow-up visits were not timely leading to a large number of cases of regorafenib termination.

### Outcomes

**Fig. 2:**
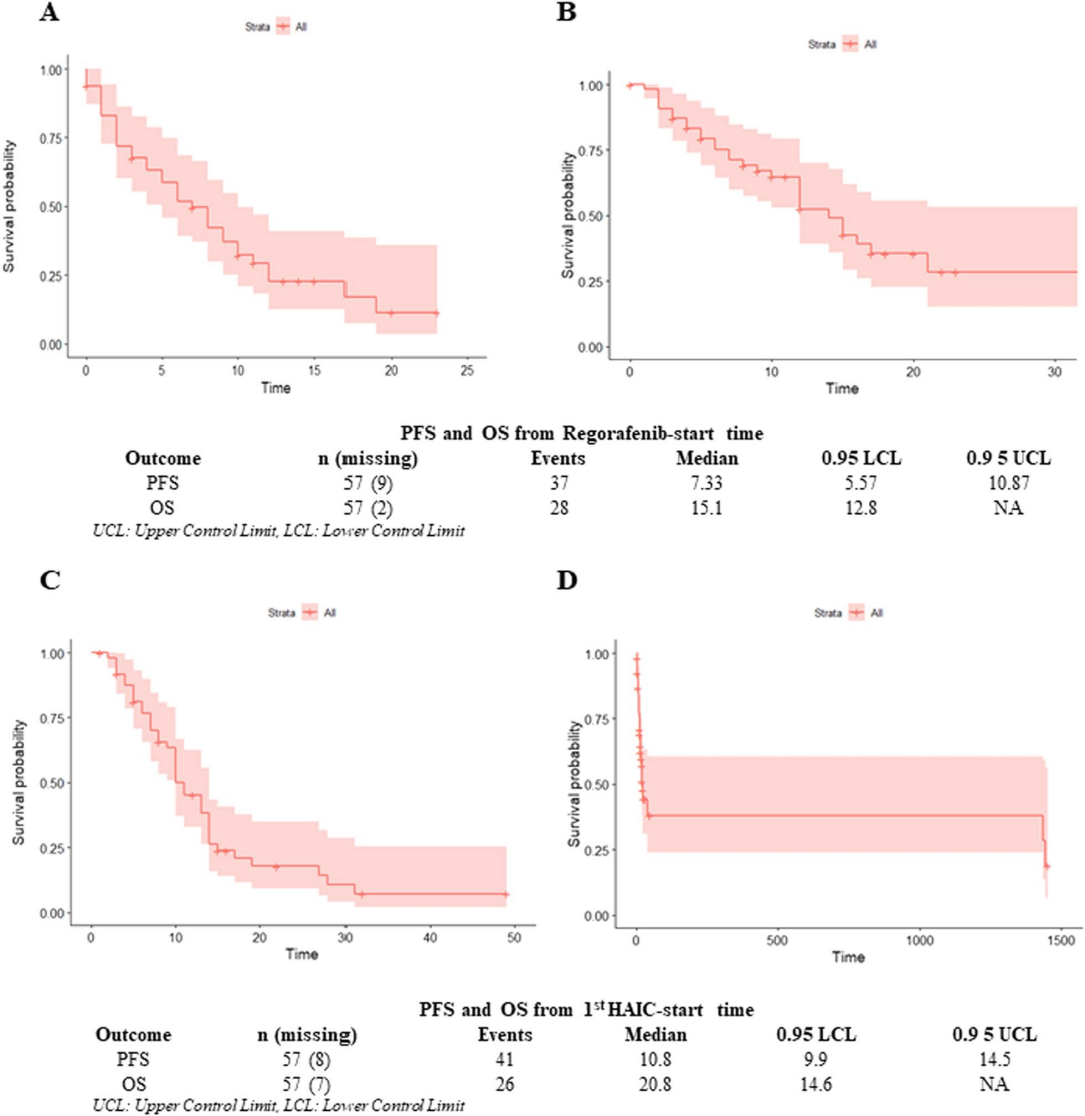
Kaplan–Meier Curves for **A** PFS from Regorafenib-start time; **B** OS Regorafenib-start time; **C** PFS from 1st HAIC-start time; **D** OS from 1st HAIC-start time. PFS, progression-free survival; OS, overall survival; HAIC, hepatic arterial infusion chemotherapy

The 1-year survival rate was found to be 64.4% (95% CI: 52.2%, 79.3%) from the start of regorafenib-start time (Fig. 2). When calculated from the 1^st^ HAIC-start time, the median PFS was 10.8 months (9.9, 14.5 months), and the median OS of 20.8 months (14.6 months, NA). The 1-year survival rate in the HAIC-start time group was 70.5% (95% CI 58.6%, 84.8%).

The best treatment responses were PR in 4 (7.0%), SD in 7 (12.3%), and PD in 7 (12.3%) when evaluated for extrahepatic complications after 1st administration of HAIC plus regorafenib, while 12 (21.1%) were categorized as others and 27 (47.4%) missing. The intrahepatic evaluation after 1st administration of HAIC plus regorafenib yielded an ORR of 50% and DCR of 100%, while the ORR was 13.3% and DCR was 36.7% among 29 patients evaluated for tumor responses were outside the liver (Table 1).

### Univariable analyses

**Fig. 3:**
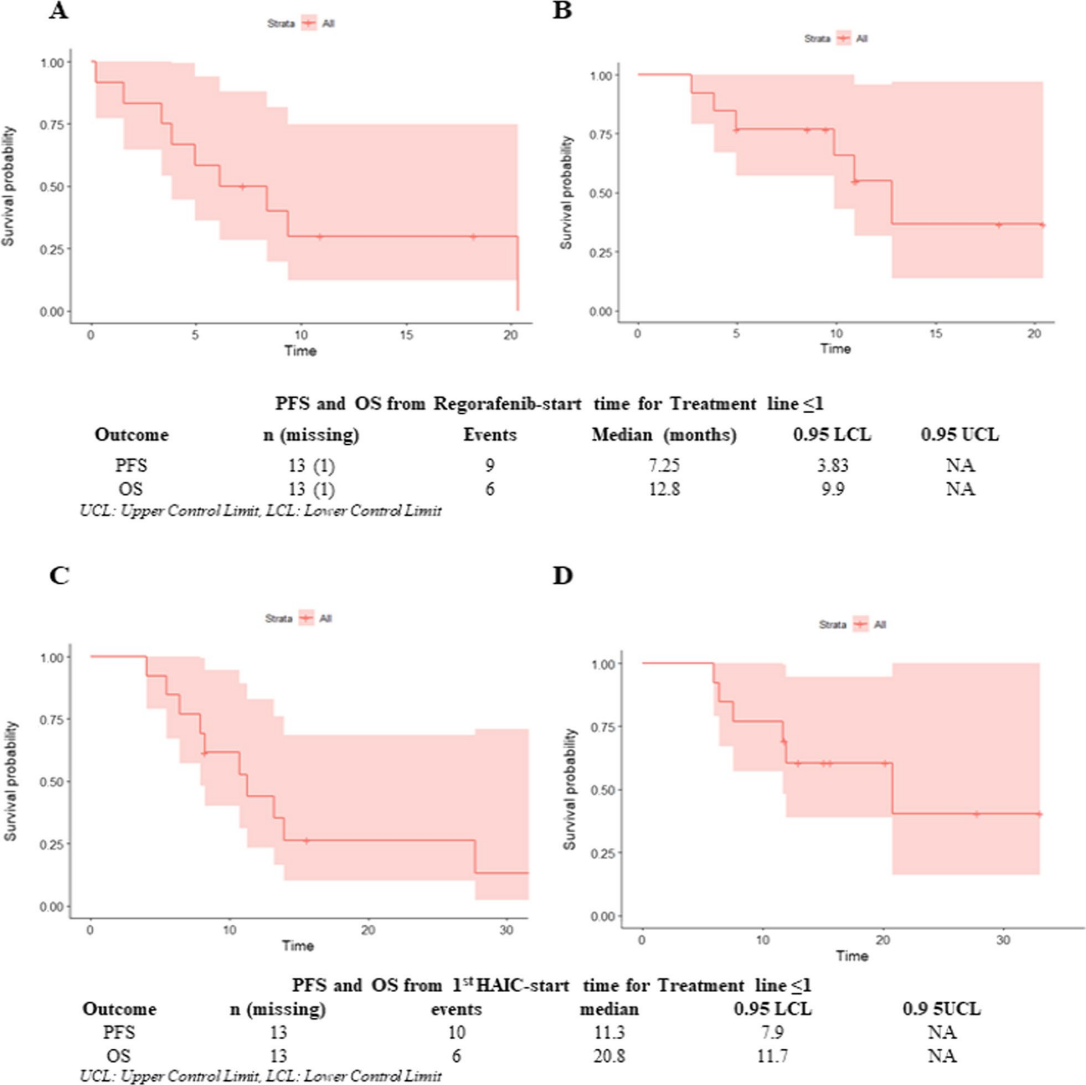
Kaplan–Meier Curves for **A** PFS from Regorafenib-start time for Treatment line ≤ 1; **B** OS from Regorafenib-start time for Treatment line ≤ 1; **C** PFS from 1st HAIC-start time for Treatment line ≤ 1; **D** OS from 1st HAIC-start time for Treatment line ≤ 1. PFS, progression-free survival; OS, overall survival; HAIC, hepatic arterial infusion chemotherapy

Cox univariable analyses were performed for PFS. Although, the starting doses of regorafenib was 40 mg/d, regorafenib dose at 120 or 160 mg/d was associated with higher PFS than 80 mg/d (log-rank *P* = 0.002; HR = 0.21, 95% CI 0.07–0.62, *P* = 0.005) (Fig. 3B, C). Patients achieving PR, or SD had a better PFS than those with PD for extrahepatic lesions (log-rank *P* < 0.001; HR = 6.0, 95% CI 2.27–15.82, *P* < 0.001) (Fig. 3D).

**Fig. 4:**
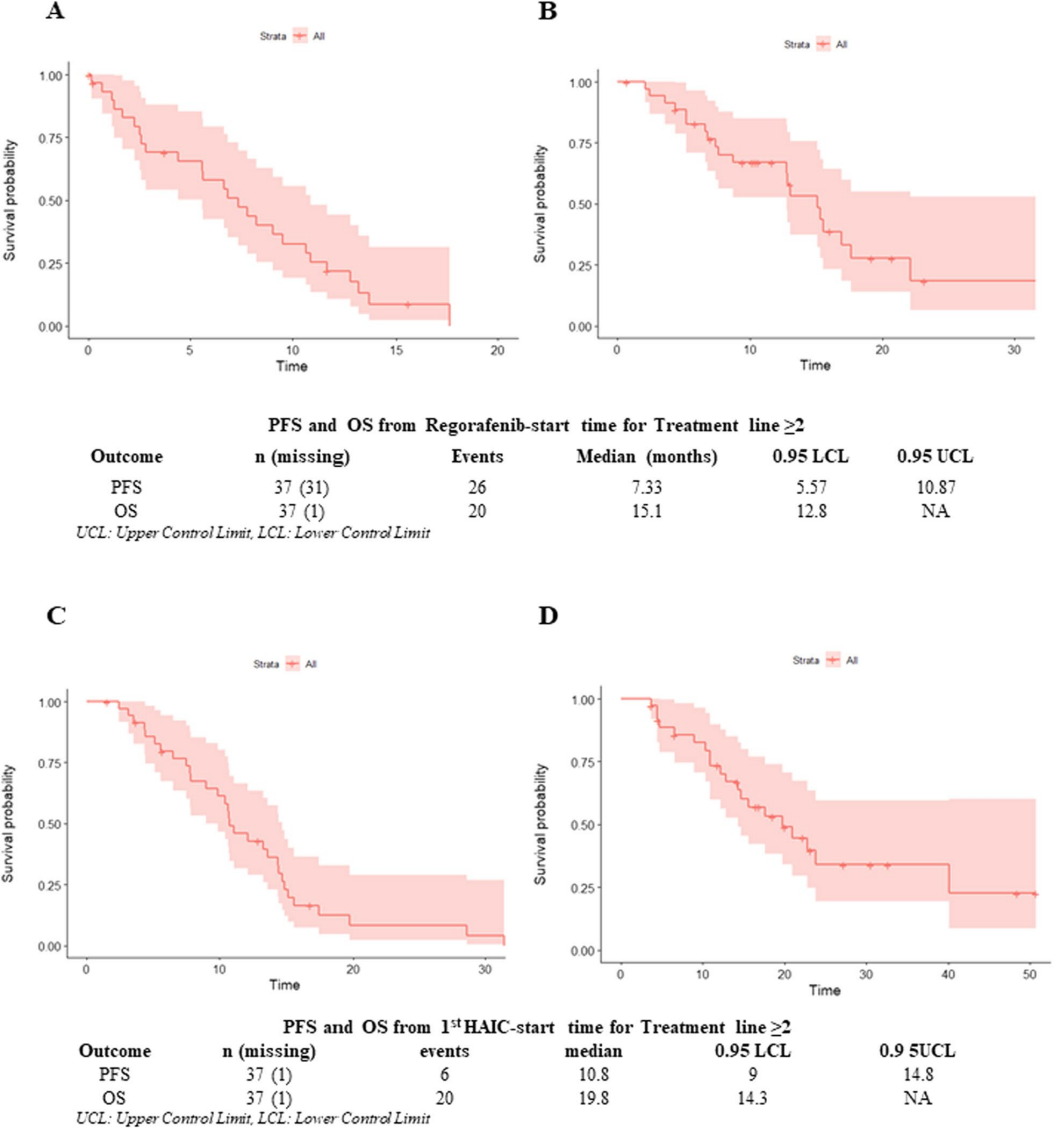
Survival analysis of **A** PFS from Regorafenib-start time for Treatment line ≥ 2; **B** OS from Regorafenib-start time for Treatment line ≥ 2; **C** PFS from 1st HAIC-start time for Treatment line ≥ 2; **D** OS from 1st HAIC-start time for Treatment line ≥ 2. PFS, progression-free survival; OS, overall survival; HAIC, hepatic arterial infusion chemotherapy

Cox univariable analysis demonstrated that higher CEA level was associated with shorter TTP than lower CEA level (log-rank *P* = 0.006; HR = 4.19, 95% CI 1.39–12.60, *P* = 0.011) (Fig. 3A). Further, involvement of large tumors was associated with shorter TTP (log-rank *P* = 0.029; HR = 3.25, 95% CI 1.08–9.82, *P* = 0.037) (Fig. 4A, B). Compared to 80 mg/d, higher dose of regorafenib at 120 or 160 mg/d was associated with longer TTP (log-rank *P* = 0.039; HR = 0.29, 95% CI 0.09–1.01, *P* = 0.051) (Fig. 4C), however the starting dose was 40 mg/d which was given to only one patient. Patients achieving CR, PR, or SD had a longer TTP compared to those with PD (log-rank *P* = 0.001; HR = 4.86, 95% CI 1.73–13.64, *P* = 0.003) (Fig. 4D).

**Fig. 5:**
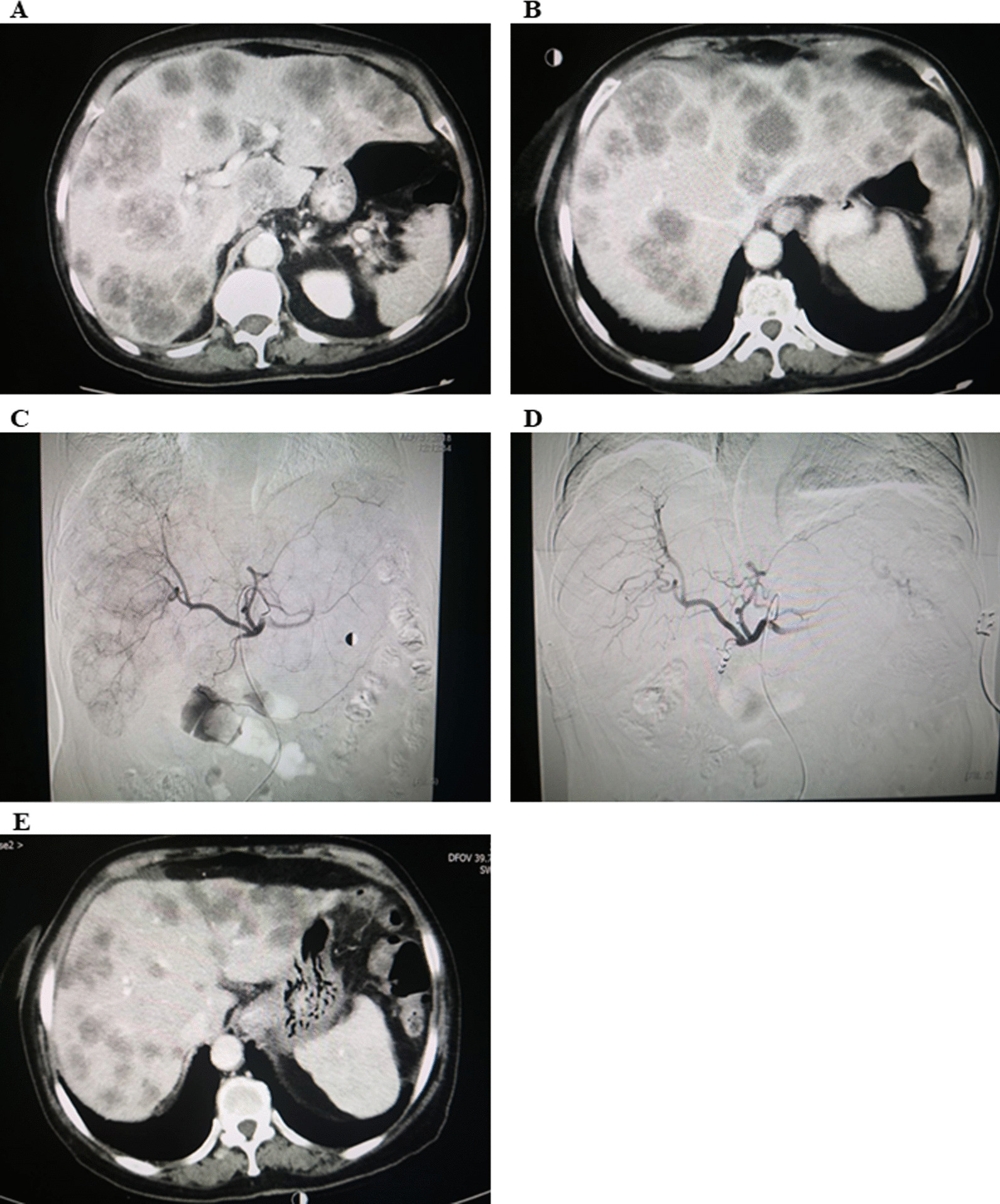
CT scans **A** diffuse lesions with 70% involvement unresected liver; **B** PD with > 1 line of systemic treatments; **C** 1st HAIC typical tumor stain illustrating vigorous lesions; **D** 2nd HAIC showed remarkable reduction in tumor stain; **E** PR achieved as per the mRECIST criteria with PFS > 6 months. CT, computer tomography; HAIC, hepatic arterial infusion chemotherapy; mRECIST, modified response evaluation criteria in solid tumors; PD, progressive disease; PFS, progression-free survival; PR, partial response

Cox univariable analyses were performed with OS as the outcome, where it was found that CEA was not associated with OS (log-rank *P* = 0.057; HR = 3.49, 95% CI 0.86–14.20, *P* = 0.081) (Fig. 5A). Similarly, tumor liver involvement was not associated with OS (log-rank *P* = 0.648; HR = 1.37, 95% CI 0.34–5.55, *P* = 0.66) (Fig. 5B). Compared to 80 mg/d, dose of regorafenib at 120 or 160 mg/d was associated with longer OS (log-rank *P* = 0 < 0.001; HR = 0.043, 95% CI 0.01–0.40, *P* = 0.006) with starting dose of 40 mg/d (Fig. 5C). Patients who achieved CR, PR, or SD had a significantly longer OS than those with PD (log-rank *P* = 0.001; HR = 9.15, 95% CI 1.82–45.94, *P* = 0.007) (Fig. 5D, E).

### Toxicity

Patients harboring AE before consumption of regorafenib, and regorafenib related adverse drug reactions (ADRs) are demonstrated in Tables 2 and 3, respectively. As shown in Table 4, no grade 5 AEs (death) were observed. Complications post HAIC were nausea (n = 11, 28.9%), pain (n = 11, 28.9%), fever (n = 4, 10.5%), and vomiting (n = 3, 7.9%). In total, 7 (18.4%) patients underwent at least one grade 3–4 AE. Two patients withdrew drug administration because of AEs. The most common AEs (≥ 10%) were hand-foot syndrome (n = 8, 21.1%), thrombocytopenia (n = 5, 13.2%), leukopenia (n = 4, 10.5%), anemia (n = 4, 10.5%), and elevated aspartate transaminase levels (n = 4, 10.5%).

**Table 2:** Laboratory examination AE of 47 patients (2 weeks before taking Regorafenib—1 month after stopping Regorafenib)

Item	Any grades		Grade 3–4		Grade 1–2	
	Number of cases	(%)	Number of cases	(%)	Number of cases	(%)
LYM	26	45.61	11	19.30	15	26.32
WBC	11	19.30	3	5.26	8	14.04
Albumin	19	33.33	0	0.00	19	33.33
Alkaline phosphatase	3	5.26	2	3.51	1	1.75
Direct bilirubin	16	28.07	6	10.53	10	17.54
Total bilirubin	17	29.82	2	3.51	15	26.32

The result of this table is the highest level of corresponding symptoms, and the percentage denominator is 47

*LYM* lymphocyte percentage, *WBC* white blood cells

**Table 3:** Regorafenib related ADRs in 47 patients

Item	Grade 3–4	
	Number of cases	(%)
HFS	2	3.51
Haemorrhage	1	1.75
Nausea	0	0.00
Dysphonia	0	0.00
Weakness	0	0.00
Diarrhoea	1	1.75
Hypertension	1	1.75
Arthralgia	0	0.00
Urinary-tract infection	0	0.00
Vomit	0	0.00
Rash	0	0.00
Upper respiratory tract infection	0	0.00
Loss of appetite	0	0.00
Lose weight	0	0.00
Stomach ache	1	1.75
Myocardial ischemia	0	0.00

The result of this table is the highest level of corresponding symptoms, and the percentage denominator is 47

*HFS* hand-foot syndrome

**Table 4:** HAIC-ADR of 47 patients

Item	Any grades		Grade 3–4		Grade 1–2	
	Number of cases	(%)	Number of cases	(%)	Number of cases	(%)
Leukopenia	1	1.75	1	1.75	0	0.00
Constipation	1	1.75	0	0.00	1	1.75
Haemorrhage	1	1.75	0	0.00	1	1.75
Nausea	29	50.88	0	0.00	29	50.88
Weakness	3	5.26	0	0.00	3	5.26
Abdominal pain	29	50.88	0	0.00	29	50.88
Abdominal distention	1	1.75	0	0.00	1	1.75
Liver injury	1	1.75	1	1.75	0	0.00
Infected	8	14.04	1	1.75	7	12.28
Myelosuppression	1	1.75	1	1.75	0	0.00
Vomit	15	26.32	0	0.00	15	26.32
Insomnia	1	1.75	0	0.00	1	1.75
Loss of appetite	3	5.26	3	5.26	0	0.00
Limb Pain	1	1.75	0	0.00	1	1.75
Elevated Serum Bilirubin (Hepatic Sinus Obstruction Syndrome)	1	1.75	0	0.00	1	1.75
Thrombocytopenia	1	1.75	1	1.75	0	0.00

The result of this table is the highest level of corresponding symptoms, and the percentage denominator is 4

## Discussion

HAIC plus regorafenib might be an appropriate recommendation for treating predominant liver metastasis of CRC after failure to a prior systemic line of treatment. Current National Comprehensive Cancer Network (NCCN) and European Society of Medical Oncology (ESMO) guidelines recommend IATs (Intra-arterial treatments) in highly specific chemo-refractory patients with predominant liver metastatic disease [20, 30]. Several IATs including HAIC was described in NCCN guidelines: (1) TACE (lipiodol and doxorubicin-eluting beads); (2) irinotecan-loaded drug-eluting beads (DEBIRI); (3) Hepatic Artery Based Therapies (HAT); (4) yttrium 90 microsphere radioembolization (RE) have been studied with predominant hepatic metastases [20]. A meta-analysis of 90 studies concluded that all four of the aforementioned techniques have similar efficacy with a minimal disparity in survival outcomes amongst the unresectable colorectal liver patients [31]. However, the exact role and timing of implementing non-extirpative local therapies in the treatment of CRLM remain controversial. Liver metastases of CRC are presumably due to the lack of blood supply and vascular anomalies, hence, the clinical outcome of TACE for patients with CRLM is expected to be improved by HAIC.

In the current study, HAIC with regorafenib provided a 1-year survival rate of 64.4%, median PFS of 10.8 months, and the median OS of 20.8 months. The survival results obtained in this study were higher than that observed in a phase 3 RESORCE study, where regorafenib monotherapy was used in advanced HCC patients after sorafenib (median PFS: 3.1 months, 95% CI 2.8–4.2; median OS: 10.6 months, 95% CI 9.1–12.1) [32]. Similarly, the survival outcomes were comparatively lower in a real-world study by Lee et.al (median PFS: 2.7 months, 95% CI 2.5–2.9; median OS:10.0 months, 95% CI 8.4–11.6) [33]. This supports the use of HAIC with regorafenib than monotherapies as longer survival outcomes were reported with HAIC and regorafenib. Three reports [34–36] in first- or second-line treatment of metastatic CRC by HAIC combined with systemic chemotherapy have demonstrated an RR between 50.6 and 55% which is similar to the findings of our study. Noteworthily, successive chemotherapeutic infusions targeted to the liver for curing metastases through the HAIC can be considered as a feasible option. In a study by Long et.al, nausea/vomiting, hypoalbuminemia, pain, anemia, and hepatic toxicity, were more frequently seen among patients who were treated with HAIC [37]. In the current study, complications post HAIC were nausea (28.9%), pain (28.9%), fever (10.5%), and vomiting (7.9%) with no grade 5 AEs and no incidence of death indicating a tolerable safety profile.

This real-world study result showed good effectiveness and tolerance of HAIC plus regorafenib regimen. There are multiple advantages of regorafenib: (1) Regorafenib is an optimal recommendation for third-line therapy in chemo-refractory patients previously treated with VEGFR and EGFR inhibitor; (2) HAIC and sorafenib/regorafenib combination has well-established its viability in the treatment of HCC treatment, which serves as the basis for HAIC combined regorafenib may have a similar rationale and mechanism in CRLM; (3) oral regorafenib for 3 weeks with a one-week interval is convenient timing for HAIC procedure and shows a relatively good tolerance; (4) incorporating regorafenib provides a follow-up option to combination with a PD-1 inhibitor, according to the reports from REGONIVO [38] trial.

To the best of our knowledge, this study is a first of its kind in exploring the feasibility, prognosis, and toxicity of HAIC combined with regorafenib for the treatment of patients with predominant liver metastasis of CRC and failure to first/second-line treatment. In real-world clinical cases, diffuse, multiple, and a high percentage of liver involvement in CRLM with disease progression that fails at least 2–3 prior standard systemic therapies prove to be problematic posing a tremendous therapeutic challenge [39]. In this research, results from the given treatment showed promising DCR, median OS, and was well-tolerated. Oxaliplatin and 5-FU l trans-arterial infusions were also the most commonly used regimen in our center for more than ten years for CRLM, advanced HCC with vascular invasion, and biliary tract cancer, with a favorable effect and safety. This is one of the most important reasons for our selection of the mFOLFOX regimen for this study. Moreover, in the previous studies at different centers [40, 41], combinations consisting of oxaliplatin, irinotecan, 5-fluorouracil, and folic acid have also proven to be efficacious with HAIC [42–45]. There might be a possible synergistic effect when oxaliplatin is administered for 2 h and then 5-fluorouracil for 22 h for three cycles during 2 continuous days [46, 47]. This could be due to the cytotoxic activity of 5-fluorouracil on the cells recovering from the mitotic inhibition exerted by oxaliplatin as they move into the S phase [46], while also reducing side effects associated with the gastrointestinal tract. In our study, there were a few Grades 3/4 side effects and almost all patients had mild anorexia and nausea. Most patients recover within a week and those patients demonstrating ORR had improved their appetite while regaining some of their lost weight.

The main limitation of this retrospective study was the small sample size and short follow-up time, making it difficult to analyze the long-term implications. Also being a single-center and observational study, it can have confounding effects on the result due to regional bias. In addition, the study is susceptible to selection bias as the number of metastatic organs was not described. We project additional prospective well-designed studies in a larger sample population that will validate our findings and contribute towards better management of CRLM.

In conclusion, the present study provides real-world evidence indicating that HAIC combined with regorafenib is beneficial and tolerable in patients with liver predominant metastasis of CRC. HAIC plus Regorafenib should be considered as an alternative for second- or subsequent lines of therapy in patients with CRLM disease showing failure on standard chemotherapy.

## Data Availability

The datasets generated and/or analyzed during the current study are not publicly available due to data confidentiality but are available from the corresponding author on reasonable request.

## References

[1] Global Cancer Statistics 2020: GLOBOCAN estimates of incidence and mortality worldwide for 36 cancers in 185 countries—Sung—2021—CA: A Cancer Journal for Clinicians—Wiley Online Library [Internet]. [cited 2021 Jun 22]. 10.3322/caac.21660. 10.3322/caac.21660 33538338

[2] 10_8_9-Colorectum-fact-sheet.pdf [Internet]. [cited 2021 Jun 22]. https://gco.iarc.fr/today/data/factsheets/cancers/10_8_9-Colorectum-fact-sheet.pdf.

[3] Vera R, González-Flores E, Rubio C, Urbano J, Valero Camps M, Ciampi-Dopazo JJ (2020). Multidisciplinary management of liver metastases in patients with colorectal cancer: a consensus of SEOM, AEC, SEOR, SERVEI, and SEMNIM. Clin Transl Oncol [Internet].

[4] Abdalla EK, Adam R, Bilchik AJ, Jaeck D, Vauthey J-N, Mahvi D (2006). Improving resectability of hepatic colorectal metastases: expert consensus statement. Ann Surg Oncol [Internet].

[5] D’Angelica MI, Correa-Gallego C, Paty PB, Cercek A, Gewirtz AN, Chou JF (2015). Phase II trial of hepatic artery infusional and systemic chemotherapy for patients with unresectable hepatic metastases from colorectal cancer: conversion to resection and long-term outcomes. Ann Surg [Internet].

[6] Scheele J, Stangl R, Altendorf-Hofmann A (1990). Hepatic metastases from colorectal carcinoma: impact of surgical resection on the natural history. Br J Surg.

[7] Sanoff HK, Sargent DJ, Campbell ME, Morton RF, Fuchs CS, Ramanathan RK (2008). Five-year data and prognostic factor analysis of oxaliplatin and irinotecan combinations for advanced colorectal cancer: N9741. J Clin Oncol Off J Am Soc Clin Oncol.

[8] Pawlik TM, Schulick RD, Choti MA (2008). Expanding criteria for resectability of colorectal liver metastases. Oncologist.

[9] Kemeny NE, Melendez FDH, Capanu M, Paty PB, Fong Y, Schwartz LH (2009). Conversion to resectability using hepatic artery infusion plus systemic chemotherapy for the treatment of unresectable liver metastases from colorectal carcinoma. J Clin Oncol Off J Am Soc Clin Oncol.

[10] Kanat O, Gewirtz A, Kemeny N (2012). What is the potential role of hepatic arterial infusion chemo-therapy in the current armamentorium against colorectal cancer. J Gastrointest Oncol.

[11] Arai Y, Ohtsu A, Sato Y, Aramaki T, Kato K, Hamada M (2012). Phase I/II study of radiologic hepatic arterial infusion of fluorouracil plus systemic irinotecan for unresectable hepatic metastases from colorectal cancer: Japan Clinical Oncology Group Trial 0208-DI. J Vasc Interv Radiol JVIR.

[12] Datta J, Narayan RR, Kemeny NE, D’Angelica MI (2019). Role of hepatic artery infusion chemotherapy in treatment of initially unresectable colorectal liver metastases: a review. JAMA Surg.

[13] de Baere T, Tselikas L, Yevich S, Boige V, Deschamps F, Ducreux M (2017). The role of image-guided therapy in the management of colorectal cancer metastatic disease. Eur J Cancer [Internet].

[14] Parmar A, Chan KKW, Ko YJ (2019). Metastatic colorectal cancer: therapeutic options for treating refractory disease. Curr Oncol [Internet].

[15] Groot Koerkamp B, Sadot E, Kemeny NE, Gönen M, Leal JN, Allen PJ (2017). Perioperative hepatic arterial infusion pump chemotherapy is associated with longer survival after resection of colorectal liver metastases: a propensity score analysis. J Clin Oncol [Internet].

[16] Long G-B, Xiao C-W, Zhao X-Y, Zhang J, Li X (2020). Effects of hepatic arterial infusion chemotherapy in the treatment of hepatocellular carcinoma. Medicine (Baltimore) [Internet].

[17] Wilhelm SM, Dumas J, Adnane L, Lynch M, Carter CA, Schütz G (2011). Regorafenib (BAY 73–4506): a new oral multikinase inhibitor of angiogenic, stromal and oncogenic receptor tyrosine kinases with potent preclinical antitumor activity. Int J Cancer.

[18] Grothey A, Van Cutsem E, Sobrero A, Siena S, Falcone A, Ychou M (2013). Regorafenib monotherapy for previously treated metastatic colorectal cancer (CORRECT): an international, multicentre, randomised, placebo-controlled, phase 3 trial. Lancet Lond Engl.

[19] Li J, Qin S, Xu R, Yau TCC, Ma B, Pan H (2015). Regorafenib plus best supportive care versus placebo plus best supportive care in Asian patients with previously treated metastatic colorectal cancer (CONCUR): a randomised, double-blind, placebo-controlled, phase 3 trial. Lancet Oncol.

[20] National Cancer Comprehensive Network. NCCN guidelines for colon cancer (version 2.2021) [Internet]. [cited 2021 Jun 23]. https://www.nccn.org/professionals/physician_gls/pdf/colon.pdf.

[21] Jiang ZC, Sun YK, Zhang W, Yang L, Cui CX, Wang HY (2020). Analysis of metastatic colorectal cancer patients treated with regorafenib in real-world practice. Zhonghua Yi Xue Za Zhi.

[22] Arnold D, Prager GW, Quintela A, Stein A, Moreno Vera S, Mounedji N (2018). Beyond second-line therapy in patients with metastatic colorectal cancer: a systematic review. Ann Oncol [Internet].

[23] Shitara K, Yamanaka T, Denda T, Tsuji Y, Shinozaki K, Komatsu Y (2019). REVERCE: a randomized phase II study of regorafenib followed by cetuximab versus the reverse sequence for previously treated metastatic colorectal cancer patients. Ann Oncol Off J Eur Soc Med Oncol.

[24] Cao F, Zheng J, Luo J, Zhang Z, Shao G (2021). Treatment efficacy and safety of regorafenib plus drug-eluting beads-transarterial chemoembolization versus regorafenib monotherapy in colorectal cancer liver metastasis patients who fail standard treatment regimens. J Cancer Res Clin Oncol.

[25] Cercek A, Boucher TM, Gluskin JS, Aguiló A, Chou JF, Connell LC (2016). Response rates of hepatic arterial infusion pump therapy in patients with metastatic colorectal cancer liver metastases refractory to all standard chemotherapies. J Surg Oncol [Internet].

[26] Liu B-J, Gao S, Zhu X, Guo J-H, Zhang X, Chen H (2020). Sorafenib combined with embolization plus hepatic arterial infusion chemotherapy for inoperable hepatocellular carcinoma. World J Gastrointest Oncol [Internet].

[27] Zervoudakis A, Boucher T, Kemeny NE (2017). Treatment options in colorectal liver metastases: hepatic arterial infusion. Visc Med [Internet].

[28] Saeki I, Yamasaki T, Maeda M, Hisanaga T, Iwamoto T, Fujisawa K (2018). Treatment strategies for advanced hepatocellular carcinoma: Sorafenib vs hepatic arterial infusion chemotherapy. World J Hepatol [Internet].

[29] Bekaii-Saab T, Kim R, Kim TW, O’Connor JM, Strickler JH, Malka D (2019). Third- or later-line therapy for metastatic colorectal cancer: reviewing best practice. Clin Colorectal Cancer [Internet].

[30] Van Cutsem E, Cervantes A, Adam R, Sobrero A, Van Krieken JH, Aderka D (2016). ESMO consensus guidelines for the management of patients with metastatic colorectal cancer. Ann Oncol Off J Eur Soc Med Oncol.

[31] Zacharias AJ, Jayakrishnan TT, Rajeev R, Rilling WS, Thomas JP, George B (2015). Comparative effectiveness of hepatic artery based therapies for unresectable colorectal liver metastases: a meta-analysis. PLoS ONE [Internet].

[32] Bruix J, Qin S, Merle P, Granito A, Huang Y-H, Bodoky G (2017). Regorafenib for patients with hepatocellular carcinoma who progressed on sorafenib treatment (RESORCE): a randomised, double-blind, placebo-controlled, phase 3 trial. Lancet Lond Engl.

[33] Lee MJ, Chang SW, Kim JH, Lee Y-S, Cho SB, Seo YS (2021). Real-world systemic sequential therapy with sorafenib and regorafenib for advanced hepatocellular carcinoma: a multicenter retrospective study in Korea. Invest New Drugs.

[34] Goéré D, Deshaies I, de Baere T, Boige V, Malka D, Dumont F (2010). Prolonged survival of initially unresectable hepatic colorectal cancer patients treated with hepatic arterial infusion of oxaliplatin followed by radical surgery of metastases. Ann Surg [Internet].

[35] Pilati P, Mammano E, Mocellin S, Tessari E, Lise M, Nitti D (2009). Hepatic arterial infusion for unresectable colorectal liver metastases combined or not with systemic chemotherapy. Anticancer Res [Internet].

[36] Mancini R, Tedesco M, Garufi C, Filippini A, Arcieri S, Caterino M (2003). Hepatic arterial infusion (HAI) of cisplatin and systemic fluorouracil in the treatment of unresectable colorectal liver metastases. Anticancer Res.

[37] Long G-B, Xiao C-W, Zhao X-Y, Zhang J, Li X (2020). Effects of hepatic arterial infusion chemotherapy in the treatment of hepatocellular carcinoma: a meta-analysis. Medicine (Baltimore).

[38] Fukuoka S, Hara H, Takahashi N, Kojima T, Kawazoe A, Asayama M (2020). Regorafenib plus nivolumab in patients with advanced gastric or colorectal cancer: an open-label, dose-escalation, and dose-expansion phase Ib trial (REGONIVO, EPOC1603). J Clin Oncol Off J Am Soc Clin Oncol.

[39] Cao F, Zheng J, Luo J, Zhang Z, Shao G (2021). Treatment efficacy and safety of regorafenib plus drug-eluting beads-transarterial chemoembolization versus regorafenib monotherapy in colorectal cancer liver metastasis patients who fail standard treatment regimens. J Cancer Res Clin Oncol [Internet].

[40] Guo J-H, Liu S-X, Gao S, Kou F-X, Zhang X, Wu D (2020). Transarterial chemoembolization with hepatic arterial infusion chemotherapy plus S-1 for hepatocellular carcinoma. World J Gastroenterol [Internet].

[41] Zhang H, Guo J, Gao S, Zhang P, Chen H, Wang X (2017). Prognostic factors for transarterial chemoembolization combined with sustained oxaliplatin-based hepatic arterial infusion chemotherapy of colorectal cancer liver metastasis. Chin J Cancer Res [Internet].

[42] Zhang H-Y, Guo J-H, Gao S, Chen H, Wang X-D, Zhang P-J (2018). Effect of primary tumor side on survival outcomes in metastatic colorectal cancer patients after hepatic arterial infusion chemotherapy. World J Gastrointest Oncol.

[43] Lévi FA, Boige V, Hebbar M, Smith D, Lepère C, Focan C (2016). Conversion to resection of liver metastases from colorectal cancer with hepatic artery infusion of combined chemotherapy and systemic cetuximab in multicenter trial OPTILIV. Ann Oncol Off J Eur Soc Med Oncol.

[44] Del Freo A, Fiorentini G, Sanguinetti F, Muttini MP, Pennucci C, Mambrini A (2006). Hepatic arterial chemotherapy with oxaliplatin, folinic acid and 5-fluorouracil in pre-treated patients with liver metastases from colorectal cancer. Vivo Athens Greece.

[45] Boige V, Malka D, Elias D, Castaing M, De Baere T, Goere D (2008). Hepatic arterial infusion of oxaliplatin and intravenous LV5FU2 in unresectable liver metastases from colorectal cancer after systemic chemotherapy failure. Ann Surg Oncol.

[46] Qin B, Tanaka R, Shibata Y, Arita S, Ariyama H, Kusaba H (2006). In-vitro schedule-dependent interaction between oxaliplatin and 5-fluorouracil in human gastric cancer cell lines. Anticancer Drugs.

[47] Takara K, Fujita M, Minegaki T, Yamamoto K, Takahashi M, Yokoyama T (2012). Treatment schedule-dependent effect of 5-fluorouracil and platinum derivatives in colorectal cancer cells. Eur J Pharm Sci Off J Eur Fed Pharm Sci.

